# Dermal Fillers and COVID-19: Angioedema With Urticaria in a Patient Post COVID-19 Infection

**DOI:** 10.7759/cureus.24461

**Published:** 2022-04-25

**Authors:** Gurnam Virdi

**Affiliations:** 1 Dermatology, Academy of Aesthetic Medicine, Sheffield, GBR

**Keywords:** corona virus, aesthetic medicine, delayed hypersensitivity, dermal filler, covid 19

## Abstract

Hyaluronic acid dermal fillers are a very popular choice for patients wanting to undergo non-invasive facial rejuvenation. Its prevalence is predicted to continue to rise. We report a case of delayed angioedema and associated urticaria to hyaluronic acid dermal fillers post COVID-19 infection. To our knowledge, this is an unusual case of hypersensitivity reaction of eight-month latency. Although the aetiology warrants further research, the author suggests utilisation of a four-week time window between dermal filler injections and COVID-19 vaccination.

## Introduction

The recent pandemic with COVID-19 has increased the prevalence of delayed complications in association with hyaluronic acid dermal fillers. We present an unusual case of hypersensitivity reaction of eight-month latency in a clinically well patient who had hyaluronic acid dermal fillers. She has had similar dermal filler treatments multiple times in the past; however, she has never reported any reactions or adverse effects.

## Case presentation

We present a case of a 55-year-old healthy female who developed a COVID-19 infection which did not require hospitalisation. She was treated with dermal fillers at multiple sites (namely cheeks, lips, jawline, and chin) for facial rejuvenation in July 2020. The hyaluronic acid dermal fillers that were injected by the clinician were monophasic in nature, 20mg/mL concentration, and crosslinked. She has been having dermal filler treatments for the past five years and never reported any reactions or adverse events. She has no medical history nor is she on any medication.

**Figure 1:**
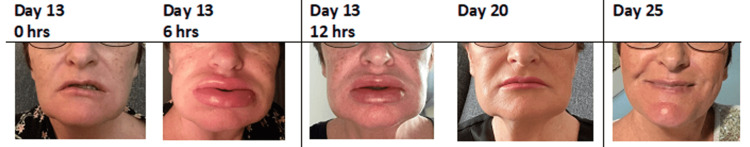
Timeline of hypersensitivity events from day 13 post-COVID-19 infection

The patient began to develop flu-like symptoms and subsequently tested positive for the COVID-19 using a polymerase chain reaction test. Surprisingly, thirteen days later she developed angioedema of the lower face and lips as well as urticaria of the hands despite not having had any dermal filler treatments for 8 months. Upon clinical examination, she was asymptomatic with regards to COVID-19 but there was firm swelling palpated at the site of the previously injected dermal fillers, except over the zygomatic region. She was managed with oral corticosteroids (60mg prednisolone with a gradual taper) and high-dose antihistamines (40mg cetirizine daily) which eventually allowed the swelling to subside. Hyaluronidase was kept on standby but was not required given the marked improvement of the swelling and tenderness in the first 24 hours. Symptoms completely resolved by day 25 without the need of dissolving the hyaluronic-acid dermal fillers with hyaluronidase (Figure 1).

## Discussion

Hyaluronic acid is generally known to be a biocompatible, non-immunogenic linear polysaccharide made from N-acetyl-d-glucosamine and glucuronic acid [1]. It has several uses including wound healing and anti-ageing. Typical delayed hypersensitivity reactions usually occur after two to four weeks post-injection with dermal fillers and may manifest in the form of erythema, oedema, and nodules [2]. In addition, certain triggers may exacerbate such responses, for example, poor injection technique, wrong product choice, dental procedures, medications, and viral illnesses [2]. It is reassuring to know that the reported global incidence rate of both immediate and delayed hypersensitivity reactions for hyaluronic acid derma fillers is only 0.8% [2]. Immediate hypersensitivity reactions typically occur within minutes via a mast-cell mediated response causing the release of histamines. On the other hand, delayed hypersensitivity reactions typically occur after a few days, although they can also occur several weeks or months later [3].

In our case, a medically well patient at the age of 55, was treated with hyaluronic acid dermal fillers for facial rejuvenation. She was not injection-naive as has never reported any acute or delayed complications in the past. With her last treatment being eight months previously, surprisingly she developed an alleged delayed reaction to the dermal fillers. A thorough clinical history revealed the likelihood of COVID-19 infection and, subsequently, she was antigen positive. What is unusual in this particular case is the presence of urticaria on the patients' hands. it is difficult to conclude the exact aetiology of this; however, one explanation could be the breakdown of hyaluronic acid into smaller fragments causing a systemic inflammatory response. It is important to consider differential diagnoses of late-onset nodules, foreign body granulomatous reactions, and biofilms.

The literature review highlighted that three patients reported facial and lip swelling after receiving the Moderna COVID-19 vaccine - two had cheek fillers 6 months prior to being vaccinated and one had lip filler two days post-vaccine [4]. Each of these patients was treated with oral corticosteroids and antihistamines. However, in our patient, the delayed hypersensitivity reaction at the site of filler injections was noted 15 days post active COVID-19 infection and not post COVID-19 vaccination. Amongst existing theories as to why delayed hypersensitivity reactions are seen in patients who have dermal fillers and certain infective viral triggers, is that dermal fillers may act as adjuvants to T-cell activation, therefore, enhancing the antigen-specific immune response [5].

The authors recommend thorough screening of patients prior to receiving the COVID-19 vaccine, for example, discussions with patients seeking cosmetic treatments who are yet to receive the vaccine. From our experience, we suggest there is a time window to minimise the risk of adverse reactions - a minimum of four weeks between dermal filler injections and vaccination in those who are deemed low risk and potentially longer in those who have risk factors, for example, immunological disorders. Furthermore, we advise that clinicians take a detailed clinical history in patients with a record of receiving hyaluronic acid dermal filler treatments as COVID-19 infection may manifest with cutaneous symptoms such as angioedema and urticaria.

## Conclusions

We presented a case of delayed hypersensitivity response to dermal fillers post COVID-19 infection. Cutaneous reactions to both the COVID-19 infection and vaccine may be an early sign of dermatologic sequelae as more of the global population gets vaccinated. The exact aetiology of delayed reactions in relation to dermal fillers and COVID-19 remains unclear and warrants further research and discussion.
